# Spanish word generation dataset from structured consonant prompts

**DOI:** 10.1038/s41597-025-05707-0

**Published:** 2025-08-11

**Authors:** Jon Andoni Duñabeitia

**Affiliations:** Centro de Investigación Nebrija en Cognición (CINC), Universidad Nebrija, Madrid, Spain

**Keywords:** Human behaviour, Human behaviour

## Abstract

This dataset captures responses from a lexical generation task designed to examine word production under structural constraints. Native Spanish speakers were presented with three-consonant strings and instructed to generate valid five-to-seven-letter Spanish words by inserting only vowels, maintaining the consonants in their original relative order. The task was conducted under time pressure and without semantic cues, allowing researchers to explore lexical access, phonotactic preferences, and the role of consonants and vowels in word formation processes. The dataset includes both item-level and participant-level files. Item-level data comprise individual responses with lexical frequency, word length, and response time. Participant-level data summarize age, gender, and aggregate lexical metrics per individual. This resource enables a range of investigations, including analyses of syllabic structures, relative consonant positioning, lexical diversity, and frequency effects. The dataset is encoded in UTF-8 CSV format and is directly compatible with standard data analysis environments. It offers a valuable tool for researchers studying lexical creativity and orthographic processing in Spanish.

## Background & Summary

Imagine being a native English speaker and being presented with a three-consonant skeleton like STR and asked to produce an English word with a length between 5 and 7 letters that incorporates these consonants in order, inserting only vowels in any desired position. Fluent speakers with an average vocabulary level might generate words like *stair, stare*, *store*, or *steer*, after rapidly searching in their mental lexicon the words that satisfy the constraints imposed by the task commands. Interestingly, while these words may be readily available in the lexicon to all speakers, the generated word would likely vary across speakers, with diverse factors determining the outcome element. Despite its apparent simplicity, this type of constrained word generation engages one of the most intricate mechanisms in human language cognition: how lexical access is guided, facilitated, and sometimes restricted by sub-lexical structures and lexical factors, such as word frequency or the age of acquisition of the words.

A growing body of psycholinguistic evidence suggests that the human cognitive system does not treat all orthographic and phonological units equally. Specifically, a large series of studies has demonstrated a functional dissociation between consonants and vowels regarding their role, a phenomenon captured by the Consonant-Vowel Hypothesis^1,2^. Consonants have been proposed as having a predominant role in lexical processing in most alphabetic languages, constraining lexical search processes and serving as the core anchors of word generation and retrieval (e.g., the Lexical Constraint Hypothesis^3^). In fact, when auditorily presented with a nonword like /kebra/ and asked to change one unit to generate a real word, individuals prefer to respect the consonantal skeleton and produce a word by replacing one of the vowels (e.g., cobra) instead of replacing one of the consonants (e.g., zebra)^4^. In contrast, vowels have been more tightly linked to prosodic and morphosyntactic processes^1^. This division of labor originally observed in artificial grammar learning tasks^5^, has received strong support from studies on word reconstruction, masked priming, eye-tracking, and neuroimaging paradigms. For instance masked priming studies have shown that a word’s consonant skeleton and their consonant-vowel structure determine the magnitude of the observed effects, with a vowel-only masked prime like OIE eliciting different priming effects on the processing of a word like OLIVE than a consonant-only masked prime like TPC on the word TOPIC^3,6,7^. Classically, as mentioned above, these differences have been accounted for by proposals discussing the differential role of consonants and vowels^8^, a different brain signature for vowel processing compared to consonant processing^9^, as well as by the lexical constraint imposed by consonants given their typically lower frequency of appearance as compared to vowels (note that in most languages the number of vowels is lower than that of consonants, and consequently they appear more often across words^3,10^).

Despite this wealth of findings, many open questions remain about how consonantal information guides lexical access, especially in word production or generation rather than comprehension. Much of the existing research has been constrained to recognition-based paradigms, which are well suited to detecting the so-called consonant-bias in word processing^11^, but less informative about generative processes and verbal creativity. The need to explore how consonantal structures shape production dynamics particularly under certain constraints points to the need for lexical generation datasets that allow for fine-grained analysis of the underlying dynamics during word elicitation. The dataset here presented aims to fill this gap by providing a large set of human-generated words under clear-cut constraints that guide lexical production based on the use of vowels to generate words from consonant skeletons.

This approach naturally connects to debates about the different role of letter identities (consonants and vowels) in letter-position coding mechanisms, and in particular, the distinction between models that posit either absolute or relative positional coding of the orthographic units. While early theories proposed that each letter occupies a fixed slot within the word (e.g., the “r” in STORE is coded as being always in position 4), more recent evidence supports a relative position coding schema by which letters are encoded relationally rather than absolutely (e.g., the letter “r” in STORE comes after the letter “t”, but not necessarily immediately after it^12^). This flexibility in positional encoding allows for the robust recognition of words even in the presence of letter transpositions (e.g., the nonword *jugde* recognized as the word *judge*^13^), suggesting that the visual word recognition system is both tolerant and predictive, relying heavily on lexical expectations. Returning to the opening example of this article, given the presentation of a string like STR, the degree to which an individual could distort the original string by adding vowels between the consonants (e.g., STAIR vs. STORE) could be informative about letter-identity and letter-position coding mechanisms.

The dataset presented in this article can be understood as a means to test the natural synthesis of these frameworks. By presenting fixed consonant sequences in a fixed internal order but allowing flexible vowel insertion, the task mirrors the logic of relative position coding while simultaneously foregrounding the role of consonants as lexical anchors. The dataset we provide stems from a lexical creativity test that captures participants’ responses to a task requiring the production of five-to-seven-letter Spanish words from a predefined set of consonant templates, presented under time pressure and without explicit semantic cues. Constrained creativity tasks allow researchers to probe the boundaries of the mental lexicon under pressure, shedding light on mechanisms of verbal fluency, word formation, and the cognitive costs associated with rule-governed generation^14^. The task was implemented in a controlled, web-based experimental environment, enabling large-scale collection of behavioral data under tightly defined phonological constraints. The resulting corpus of thousands of elicited words, each generated under the same structural template, offers an opportunity to examine how the cognitive system uses consonantal scaffolding to construct lexical items under time-limited conditions.

In sum, the development of this dataset was motivated not only by a theoretical need to integrate models of orthographic structure and lexical access, but also by a practical need for rich, open resources that support empirical testing of these models in production settings. The dataset offers a new lens through which to investigate some of the foundational building blocks of language, and how they shape, limit, and enable the ability to generate words from structure alone.

## Methods

### Participants

A total of 480 native Spanish speakers (198 females, 268 males, and 14 persons self-identified as non-binary) took part in the data collection, with a mean age of 33.22 years (standard deviation: 10.93; range: 18–69). Participants were recruited through Prolific (www.prolific.com), an online platform known for providing high-quality and diverse research samples. The inclusion criteria that participants had to meet to take part in the study were: 1) being at least 18 years old, 2) being native Spanish speakers, and 3) living in Spain at the time of testing. Once participants had provided informed consent to participate in the Prolific platform, including explicit permission for their anonymized data to be shared in open repositories, they were redirected to the testing platform, created in Gorilla Experiment Builder^15^ (www.gorilla.sc). They were then asked to complete the task, after completing a brief demographic questionnaire. To verify the inclusion criteria, this initial questionnaire asked participants to indicate their age, sex and Autonomous Community in which they currently resided; this last item was used exclusively as a screening field to confirm eligibility and, because it was not relevant to any subsequent analyses and could increase the risk of indirect re-identification, it was not retained in the shared dataset. Upon completion of the task, participants were compensated with £10.56 per hour of dedication. The experimental procedure was approved by the Research Ethics Committee at Nebrija University (approval code: UNNE-2022-0017).

### Materials

The stimuli consisted of a curated set of 99 unique three-consonant strings (e.g., MNT) derived from a database of five-to-seven-letter Spanish words obtained from the EsPal lexical database^16^. The initial database was filtered to include only the words with exactly three consonants (e.g., MENTA, the Spanish for *mint*). From this selection, the consonant strings were extracted for each word (e.g., MNT from MENTA). The final set of 99 strings yielded a total of 3,991 possible Spanish 5-to-7-letter words, with an average Zipf lexical frequency of 2.12 (SD = 1.12, range: 0.51–6.28). The Zipf score is a standardized log-transformed measure of word frequency based on Zipf’s Law^17^, and it is currently the gold-standard to report lexical frequency^18^. The 99 final consonant strings were selected following a series of criteria imposed to guarantee variability in the user-generated outcomes. First, each string was present in at least 12 valid Spanish words of the target length values in the database, with a mean of 40.31 possible words per string (SD = 18.58; range: 12–93). And second, all the selected strings could elicit at least 4 valid Spanish words with a high Zipf frequency, with the threshold being set at a value equal to or higher than 4.

### Procedure

Participants accessed the study through the online interface and were first presented with the instructions. For each trial, they were asked to type in the designated space a valid five-to-seven-letter Spanish word that included the given consonants, allowing for the insertion of vowels only, and keeping the relative order of the consonants intact (e.g., MENTA [*mint*] or MINUTO [*minute*], among others, for the string MNT). Each response had to be submitted within a time limit of 20 seconds. Once the response had been entered, participants had to press the Enter key on the keyboard or click a button on the screen to finish the trial and move to the next one. Participants were given the choice to skip a string at any time if no words came to their mind, and they were instructed to avoid using non-existent Spanish lexemes. The experimental session started with two practice trials that preceded the test phase. In the test phase, participants were presented with a series of individual trials in which the 99 consonant strings were displayed in a random order. An internal timer was activated at the start of the test phase to measure task duration, and the task automatically ended after exactly 4 minutes. All the experimental materials and procedures are available through https://app.gorilla.sc/openmaterials/1024192 to favor reproducibility.

## Data Records

The complete dataset generated in the study is available in a publicly accessible repository^19^. The data is provided in two CSV files corresponding to the item-level and participant-level information.

The data file containing the item-level information is named RESPONSE DATA.csv and includes the columns in UTF-8 encoding described in Table 1. To prevent certain lexical tokens from being auto-coerced into Boolean values in common analysis environments, every entry in the STRING (prompt) and RESPONSE (participant answer) columns is explicitly wrapped in double quotation marks. This formatting forces all values to be imported as character strings, ensuring seamless, platform-independent processing of the data set. The FREQUENCY and RT columns use commas as decimal separators; users working in environments that expect periods (e.g., U.S. settings) should set the appropriate locale or convert the commas to periods during import so that these variables are read as numeric.

**Table 1:** Description of the content of the RESPONSE DATA.csv file.

Column Name	Description
PARTICIPANT	An anonymized unique identifier for each participant
STRING	The 3-consonant letter string that served as a prompt
RESPONSE	The letter string generated by the participant
FREQUENCY	The Zipf lexical frequency of the response
LENGTH	The length (in number of letters) of the response
RT	The response time (in milliseconds) needed to generate the response

The data file containing the participant-level information is named USER DATA.csv and includes the columns in UTF-8 encoding described in Table 2.

**Table 2:** Description of the content of the USER DATA.csv file.

Column Name	Description
PARTICIPANT	An anonymized unique identifier for each participant
AGE	The age of the participant as self-reported in years
GENDER	The gender of the participant (Male, Female, Non-binary)
NUMBER_RESPONSES	The number of valid responses generated matching the prompt string
MEAN_FREQUENCY	The mean Zipf lexical frequency of the valid responses
MEAN_LENGTH	The mean length (in number of letters) of the valid responses
MEAN_RT	The mean reaction time (in milliseconds) of the valid responses

## Technical Validation

To ensure the robustness, reliability, and scientific utility of the dataset^19^, we implemented a multi-layered validation process encompassing response verification, and global data consistency checks. First, a response-level validation was carried out. Every submission was programmatically cross-referenced with the master list of accepted words. The validation criteria required that each response contain five to seven letters, include only the consonants from the original prompt in the correct order, and use vowels as fillers in line with the defined task constraints. Critically, only words already included in the predefined ESPaL-derived database were considered valid. This automated scoring process was further complemented by a manual review of a subset of responses to ensure that the rule-based validation had been implemented correctly and consistently, thereby maximizing scoring reliability. To this end, a full manual audit was performed on the complete data sets of the first three participants, and each response was re-coded for validity. Agreement between the manual judgments and the rule-based categorization was 100%, and no additional hand checking was deemed necessary.

Across the 480 participants, a total of 13,231 five-to-seven-letter words derived from the selected consonant strings were recorded. Out of these, 12,640 responses corresponded to words that were listed in the word database, and these responses constitute the final dataset. Thus, 95.53% of all responses matched known items in the original word list, indicating a high degree of participant engagement and compliance with task instructions.

**Fig. 1:**
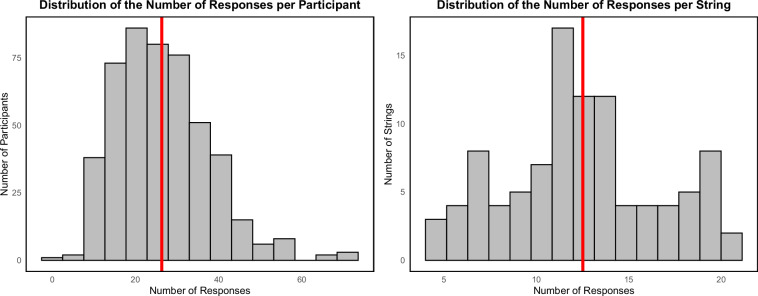
Left: Histogram showing the distribution of the total number of valid responses across participants. The red vertical line marks the mean number of responses. Right: Histogram showing the distribution of the total number of valid responses across prompt strings. The red vertical line marks the mean number of responses.

Second, a descriptive analysis was carried out to ensure that participants were generally capable of producing valid word forms under time constraints and restrictive rules. On average, participants produced 26.33 valid words (SD = 11.44; see Fig. 1, left chart, for a distribution of the number of responses). The mean length of the outcome words was 5.36 (SD = 0.15). In general terms, the Zipf frequency of the generated words was markedly high, with a mean of 4.06 (SD = 0.26). An additional descriptive analysis confirmed that the consonant strings used as prompts elicited both adequate variety and overlap in participants’ responses. On average, each string generated 12.52 distinct valid words (SD = 4.06; see Fig. 1, right panel, for the distribution of responses).

## Usage Notes

The dataset is structured to facilitate flexible re-use in studies examining word production, orthographic processing, and lexical retrieval in Spanish. Each response entry includes a fixed consonant sequence and a participant-generated word, along with lexical frequency and response time data, enabling detailed analyses of word formation under structural constraints. The dataset supports investigations into the emergence and distribution of different syllabic templates (e.g., CV, CVC, CCV) as participants insert vowels to complete the consonantal skeletons. Researchers can analyze how specific orthographic and phonotactic patterns emerge, and whether certain syllable structures are preferred over others across consonant contexts.

In addition, the design of the task allows for fine-grained exploration of the relative position of consonants within the generated words. While all items preserve the sequence order of the consonants, their precise placement within the word (e.g., word-initial vs. medial vs. final) varies across responses. This makes it possible to examine positional flexibility and constraints in lexical generation, and to model how consonantal placement influences word accessibility or production success.

The dataset includes participant-level information such as age, gender, and aggregate response metrics, which supports subgroup analyses and the investigation of individual differences in lexical diversity, fluency, and strategy use. All files are provided in UTF-8 encoded CSV format and are compatible with standard data analysis tools without requiring preprocessing.

## Data Availability

All software tools used for data processing and analysis in this study are detailed in the *Methods* section, including software versions where applicable. Analyses were conducted using standard functions and default parameters unless otherwise specified. No custom code was developed or required for the processing or analysis of the dataset.
